# Utility of the “floating ball sign” in diagnosis of ovarian cystic teratoma

**DOI:** 10.4274/tjod.galenos.2019.67209

**Published:** 2019-07-03

**Authors:** Hilal Şahin, Aslı Irmak Akdoğan, Duygu Ayaz, Tuğba Karadeniz, Muzaffer Sancı

**Affiliations:** 1 University of Health Sciences, Tepecik Training and Research Hospital, Clinic of Radiology, İzmir, Turkey; 2 University of Health Sciences, Tepecik Training and Research Hospital, Clinic of Pathology, İzmir, Turkey; 3 University of Health Sciences, Tepecik Training and Research Hospital, Clinic of Gynecologic Oncology Surgery, İzmir, Turkey

**Keywords:** Dermoid cyst, magnetic resonance imaging, tomography, ovary

## Abstract

**Objective:**

To evaluate the incidence of the “floating ball” sign in cross-sectional imaging modalities in patients with ovarian teratoma, and to investigate the relationship between the morphologic features of the teratoma and this sign.

**Materials and Methods:**

Preoperative computed tomography and magnetic resonance imaging studies of 112 women with a pathologic diagnosis of ovarian mature cystic teratoma (MCT) were reviewed for the presence of the floating ball sign. Tumor size, tumor characteristics and the number, size, and characteristics of floating globules were evaluated.

**Results:**

In 112 patients (mean age 35.5±16 years, range 5-84 years), 118 ovarian MCTs were diagnosed pathologically. The floating ball sign was demonstrated in cross-sectional imaging in 30 (25.4%) patients. Among 2 of them, MCT was associated with malignancy (squamous cell carcinoma). There was a significant relationship between the floating ball sign, tumor size, and the wall thickness of the tumor (p=0.003 and p=0.018, respectively). In linear regression analysis, a similarly significant relationship was found between the presence of this sign and tumor size and patient age (p=0.003 and p=0.035, respectively).

**Conclusion:**

The floating ball sign, as a pathognomonic sign for the diagnosis of ovarian teratomas, seems to be more common than is known. Although this sign is almost always seen in MCTs, it may rarely be seen in teratomas with malignant transformation. The relationship of this sign with the characteristics of the tumor can provide an insight into the occurrence of these balls.

**PRECIS:** We have evaluated the incidence and relationship of floating ball sign in ovarian mature cystic teratomas with patient age and tumor characteristics.

## Introduction

Mature cystic teratomas (MCTs) are the most commonly seen germ cell tumors of the ovary^(1)^. They account for at least 10% of all ovarian tumors^(2)^.They arise from two or three primitive embryonic germ cell layers, known as the endoderm, mesoderm, and ectoderm^(3)^. When the ectodermal component predominates, they are called dermoid cysts^(2)^.

Preoperative diagnosis is important because teratomas are the most common surgically excised ovarian tumor group. Imaging modalities such as ultrasonography, computed tomography (CT), and magnetic resonance imaging (MRI) help us to evaluate a wide spectrum of radiologic presentations and make the correct diagnosis^(4)^. Although ultrasound is the first-line imaging modality in the evaluation of these tumors, diagnosis sometimes might be difficult due to the limitless combinations of different echo patterns in MCTs. Cross-sectional imaging modalities are preferred in indeterminate cases or for confirmation of teratoma suspicion in ultrasound.

Fat content is the most important feature of the teratoma, which can be readily demonstrated using cross-sectional imaging. Besides that, some pathognomonic features enable the diagnosis easily. The “floating ball” sign is an uncommon pathognomonic feature that defines one or more small spherical structures floating in a cyst^(5)^. It may also be called the “meat ball” sign or the “truffle sign,” referring to numerous small floating globules, or “pokeball” sign, referring to a single ball floating in a fat-fluid interphase^(6,7)^. The latter is a recent description, which is associated with the current pop culture craze regarding Pokemon balls^(8)^.

The aim of this retrospective single center study was to assess the utility of the floating ball sign in the diagnosis of ovarian teratoma and to investigate the relationship between this rare sign and tumor morphologic features.

## Materials and Methods

### Patients and study setting

This retrospective study was approved by the University of Health Sciences, İzmir Tepecik Training and Research Hospital Ethics Committee (25^th^ April 2018, no: 2018/4-9). Patients with ovarian teratoma who were surgically treated at a single tertiary care institution between August 2009 and January 2018 were reviewed. The pathology results were collected from patients’ hospital records. The inclusion criteria of the study were as follows: a) All patients would have a pathologic diagnosis of teratoma originating from the ovary, b) patients would have undergone diagnostic abdominal CT or MRI before surgical resection performed with a standard protocol. After exclusion of two patients with immature teratomas and one patient with a mixed germ cell tumor including a teratoma component, a total of 112 patients with a diagnosis of MCT were enrolled in the study. All patients were diagnosed and treated in the same tertiary hospital with a gynecologic oncology department serving as a cancer center for gynecologic malignancies.

### Imaging protocol

MRI examinations were performed with standard protocol using a 1.5 T MRI system (Siemens Avanto, Siemens Aera, GE Optima360) with a six-channel body coil. The protocol included sagittal, axial, and coronal T2-weighted images without fat saturation, axial T2-weighted fat saturated images, and axial T1-weighted fat saturated gradient-echo images before and after intravenous contrast administration (Gadoteric acid, Dotarem^®^, Guerbet, Paris, 0.1 mmol/kg).

CT examinations were performed using a 64 and 128 detector CT system in the venous phase after intravenous non-ionic contrast administration.

### Data analysis

Images were re-evaluated by a radiologist (AIB) who was blinded to the clinical data, results of surgery, and pathology reports. The floating ball sign was defined as an appearance of a globular structure floating in a cyst without a direct relationship with the wall of the tumor. Radiologic image evaluation included the assessment of the tumor size, the thickness of the wall of the cystic mass, the presence of any globular structures floating in the cystic mass (floating ball sign), and the number and size of the floating balls. Tumor diameters were measured in three orthogonal planes and the maximum diameter was recorded as the largest tumor size. The presence of fat in the tumor was noted. To evaluate fat in MRI, both fat suppression techniques and chemical shift imaging including in-phase and opposed-phase imaging were used. In cases with the floating ball sign, the size and number of the balls, location of the balls in the cyst (gravity-dependent, gravity-independent or on a fat-fluid interphase), and the density of the balls and the presence of detectable fat in the balls were recorded.

### Surgical evaluation

All patients underwent laparotomy and frozen section assessment for the diagnosis. Fertility-sparing surgery or hysterectomy-salpingoopherectomy was performed according to the patient’s age, desire for fertility, and other circumstances such as multifibroid uterus. Pelvic lymph node dissection was performed as a part of the routine surgical procedure in 2 patients with malignant neoplasia in frozen sections. Tumors were diagnosed by clinical pathologists with more than 10 years’ experience in gynecologic pathology.

### Statistical Analysis

Statistical analyses were performed using SPSS version 20.0 (Chicago, IL, USA). Data are presented as mean ± standard deviation. The Mann-Whitney U test and Pearson’s chi-square test were applied to evaluate the relationship between the floating ball sign, and continuous variables such as patient’s age, tumor size, and tumor wall thickness. Linear regression analysis was performed to evaluate the relationship between the floating ball sign and each variable. P values were considered significant at <0.05.

## Results

### Sample characteristics

Between August 2009 and January 2018, preoperative radiologic imaging via CT or MRI was performed in 115 patients with pathologic diagnoses of ovarian teratoma. After the exclusion of two patients with immature teratomas and one patient with a mixed germ cell tumor including a teratoma component, a total of 112 patients with a diagnosis of MCT were enrolled in the study. Among them, 108 patients had pure MCTs. Two patients had a combination of MCT with another tumor (1 fibrothecoma and 1 granulosa cell tumor) and two patients had squamous cell carcinomas arising from an MCT. Six patients had teratomas in both ovaries.

The mean age of the 112 patients included in the study was 35.5±16 (median: 34, range 5-84). There were fourteen patients in the pediatric age group in the study. Among the cross-sectional imaging modalities, 29 (25%) patients were evaluated with only CT, 75 (66.9%) patients with only MRI, and 8 (7.1%) patients with both before undergoing surgery.

### Tumor characteristics

In the study, 118 ovarian MCTs were evaluated in 112 patients. Among them, malignancy (squamous cell carcinoma) was found in only 2 (1.7%) of the MCT cases in histopathologic evaluation. The mean maximum tumor size was 83 (median: 72, range 14-220) mm. The maximum wall thickness of the tumor was 3.5 (median: 1.8, range 0.7-35) mm. Fat was detected with CT or MRI in 98.3% of the MCTs.

### Floating ball sign

**Figure 1:**
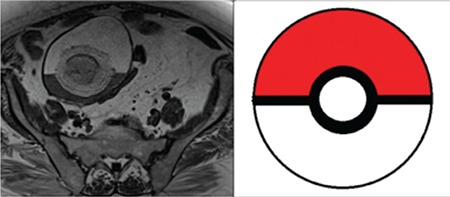
A 67-year-old woman with right ovarian mature cystic teratoma. A large floating ball is seen in the interphase of fat-fluid level in T1-weighted magnetic resonance imaging. It seems similar to the schematic drawing of “Pokemon ball” with different signal intensities of fluid, fat and the ball. Note that the ball has a central nidus as in the schematic drawing

**Figure 2:**
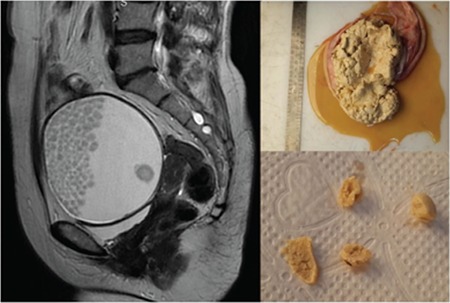
A 56-year-old woman with right ovarian mature cystic teratoma. Numerous small floating balls are seen in the gravityindependent side of the cyst. A larger floating ball is seen in the gravity-dependent side. These balls were composed of pale yellow keratinoid material in macroscopy images

Floating ball(s) were demonstrated in 30 (25.4%) MCT lesions (Figure 1 and 2). In 2 cases with the floating ball sign, MCT was associated with malignancy (squamous cell carcinoma). There was only one floating ball in half of the lesions (n=15). The maximum size of the floating balls ranged between7 and 56 (mean: 31) mm. The locations of the floating balls were as follows: 33.3% at the gravity-dependent side, 20% at the gravity-independent side, and 43.3% at the interphase of the fat-fluid level.

**Figure 3:**
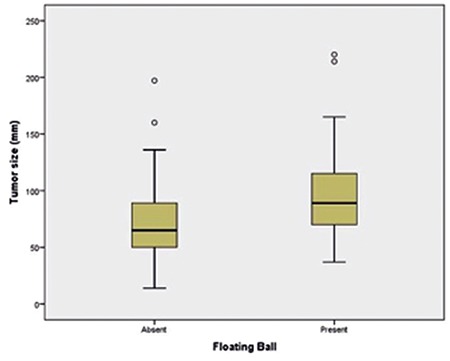
Box-and-whisker plots show maximum tumor size in the two groups with and without floating balls inside the cystic teratoma. Central horizontal line in box represents mean value, bottom and top edges of box indicate 25^th^ and 75^th^ percentiles, and vertical line indicates the range of data

In CT imaging, the density of the floating balls ranged between -70 and +35 Hounsfield units (HU) (mean -30 HU) regarding fat content. In MRI, in 64% of balls, a fat signal was demonstrated in fat suppression sequences. Overall, fat was demonstrated via CT or MRI in 66.6% of balls. As expected, most (96.7%) of MCTs with the floating ball sign had fat in the tumor; fat was not demonstrated radiologically or pathologically in the tumor in only one lesion (3.3%) with a floating ball. The characteristics of the teratoma groups with and without the floating ball sign are summarized in Table 1. The distribution of maximum tumor size in these two groups is shown in Figure 3.

### Relationship of the floating ball sign with patient’s age, tumor size, and tumor wall thickness

There was a significant relationship between the floating ball sign and the tumor size and wall thickness of the tumor (p=0.003 and p=0.018, respectively). Although the mean age of the patients was higher in the group with the floating ball sign, the relationship between this sign and patient age was not statistically significant (p=0.078). In multivariate regression analysis including those variables, there was a significant relationship between the sign, tumor size, and age (p=0.003 and p=0.035). However, the relationship was not significant regarding tumor wall thickness (p=0.663). Also, the presence of a floating ball was not significantly related to the presence of fat in the tumor (p=0.421).

## Discussion

In the present study, we evaluated the incidence of the floating ball sign in our patient cohort with ovarian teratoma and investigated the relationship between this sign and tumor morphologic parameters and patient age. Our results indicate that, this sign, with a 25% incidence rate, is much more common in clinical practice than it has been reported in the literature. Floating balls in teratomas are mentioned in case reports or case series; however, the exact incidence rate of this sign is not given in the studies. As described in some of those reports, we found that this sign was usually seen in larger MCTs and in those with thicker walls. This may give some clues about the formation of these ball-like structures. Interestingly, in our series, both MCTs with squamous cell carcinoma had the floating ball sign, which has not been mentioned in the literature until now.

Atypical presentations of ovarian teratomas can be a challenge both for radiologists and gynecologists. Thanks to the fat content, classic ovarian teratomas are easily diagnosed with imaging. However, when there is no fat or it is very scarce in amount, other radiologic features, each reflecting a specific pathologic equivalent, enable the correct diagnosis^(4,9)^.

The floating ball sign is described as a pathognomonic sign for the diagnosis of ovarian MCTs^(5,10)^. It was first described in 1991 by Muramatsu et al.^(11)^ with radiologic imaging. Later, many case reports were written on this sign regarding its rarity. However, our study shows that, with a 25% incidence rate, it is not rare, contrary to popular belief. In addition, it can be easily recognized by any physician in ultrasound, CT or MRI; therefore, it may become an important sign of MCTs and may have an educational value. Besides that, in the presence of this pathognomonic sign, abbreviated MRI protocols with shorter scan duration and without intravenous contrast agent might be sufficient for the diagnosis.

Floating balls are spherules composed of variable proportions of keratin, fibrin, hemosiderin, sebaceous material, hair, and fat^(5)^. According to the contents, these floating spherules take a gravity-dependent or gravity- independent position in the cyst. In our study, most of the balls were located at the fat-fluid interphase. Although the mechanism of the formation of those balls is still unknown, it is speculated that, globules form by aggregation of sebaceous material around a nidus made up of debris, squames or hair shafts, while moving in the cystic cavity^(5,12)^. They form into discrete spherical masses because of the difference in physical and thermal properties of the material being deposited^(13)^. In our study, the floating ball sign was significantly related to tumor size and it was seen in somewhat larger cysts. This correlates with evidence that enough space is essential for floating balls to form their globular shape^(5,14)^. In addition, as Al Hilli et al.^(15)^ proposed, the predominance of a large secretory and absorptive surface lining the cyst may favor the absorption of most of the contents causing the remaining material to solidify and mold into balls. Another variable related to the floating ball sign was the tumor wall thickness in the univariate analysis. Given that these balls are made up of keratin and sebaceous material, a thick tumor wall containing skin derivatives such as sebaceous glands may provide these materials in sufficient amounts. 

We also investigated the relationship between patient age and the floating ball sign. Although the mean age of the patient group with the sign was higher, the relationship was not significant in the univariate analysis. However, in the multivariate analysis, age was significantly related to the floating ball sign. MCTs are supposed to grow at an average rate of 1.8 mm each year according to the relevant literature^(1)^. Our result of the linear regression analysis may be related to that, as we expect larger tumors with increasing age. In addition, it is believed that small bowel peristaltism against the cyst wall encourages the viscostatic aggregation of the sebaceous material and promotes the formation of floating balls; therefore, time is needed for these balls to take their uniform, globular shape^(6)^.

Another interesting finding of our study was that there were two patients with squamous cell carcinomas growing in MCTs, both of which contained floating balls. Otigbah et al.^(7)^ claimed that the presence of hairy balls suggesting papillary projections on ultrasound might indicate a higher likelihood of malignancy; however, no related studies were found in the literature to support this proposal. According to the literature, whether globule formation occurs in both benign and malignant cysts is not known, but all cases reported thus far have been benign^(14)^. We cannot make a general statement about floating balls in malignant teratomas due to the insufficient number of malignant cases in the study for a statistical analysis. However, we can claim that, floating balls are not exclusively seen in benign cystic teratomas; therefore, the floating ball sign cannot be pathognomonic for benign teratomas. This is an important result of our study, challenging some other reports in the literature^(6,16)^.

### Study Limitations

There are a few limitations in the present study. First, it was a single-center retrospective study. Second, we did not include immature teratomas and mixed germ cell tumors in the study because there were only 3 cases. In addition, according to the relevant literature, the appearance of multiple floating spherules has not been found in tumors other than cystic teratomas^(17)^. Third, CT or MR images of the patients were assessed in the study. We believe this limitation did not cause a significant impact because the floating ball sign and fat content is easily detected in both imaging modalities. In addition, ultrasound imaging could not be compared with sectional imaging modalities due to the retrospective nature of the study. The floating ball sign was not properly noted in ultrasound reports. Lastly, the small number of malignant teratomas in the study caused a relative limitation because we could not make a statistical analysis and compare that group with benign teratomas.

## Conclusion

The floating ball sign is a popular pathognomonic sign for cystic teratomas and it is much more common in clinical practice than is known. It is seen in older patients with larger teratomas. In addition to being easily assessed in every imaging modality by any physician interested in gynecologic imaging, it is of paramount importance in the diagnosis of ovarian cystic teratoma. Nevertheless, this sign can be seen in both benign and malignant MCTs. Therefore, each teratoma lesion including floating balls should be carefully evaluated for signs of malignancy.

## Figures and Tables

**Table 1:**
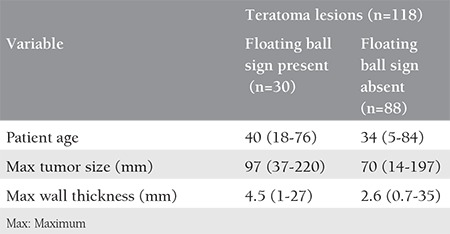
Summary of patient age, maximum tumor size, and maximum tumor wall thickness in the teratoma groups with and without the floating ball sign. Results are given in mean value (range minimum-maximum)
